# Parent- and therapist-rated treatment satisfaction following routine child cognitive-behavioral therapy

**DOI:** 10.1007/s00787-020-01528-1

**Published:** 2020-04-18

**Authors:** Paula Viefhaus, Manfred Döpfner, Lydia Dachs, Hildegard Goletz, Anja Görtz-Dorten, Claudia Kinnen, Daniela Perri, Christiane Rademacher, Stephanie Schürmann, Katrin Woitecki, Tanja Wolff Metternich-Kaizman, Daniel Walter

**Affiliations:** 1 Department of Child and Adolescent Psychiatry, Psychosomatics and Psychotherapy, Medical Faculty, University of Cologne, Robert-Koch-Str. 10, 50931 Cologne, Germany; 2 School of Child and Adolescent Cognitive Behavior Therapy (AKiP), University Hospital Cologne, Pohligstr. 9, 50969 Cologne, Germany

**Keywords:** Treatment satisfaction, Routine treatment, Differential effects, Cognitive-behavioral therapy, Children

## Abstract

This observational study examined treatment satisfaction (TS) following routine outpatient cognitive-behavioral therapy (CBT) in a large sample of children (*n* = 795; aged 6 to 10 years). TS was investigated in parent and therapist rating. Means, standard deviations and inter-rater correlations were calculated to investigate TS. Regression analysis was conducted to examine potential correlates of TS (patient-related variables, mental disorder characteristics, socio-demographic factors and treatment variables). High TS in parent and therapist rating was found, with therapists showing a lower degree of TS than parents (completely or predominantly satisfied: parent rating 94.1%, therapist rating 69.5%). A statistically significant, moderate inter-rater correlation was found. Regression analysis explained 21.8% of the variance in parent rating and 57.2% in therapist rating. Most of the TS variance was explained by mental disorder characteristics (parent-rated symptoms and therapist-rated global impairment at treatment end) and by treatment variables (especially the therapist-rated cooperation of parents and patients), whereas socio-demographic and patient-related variables did not show any relevant associations with TS. Based on these results, to optimize TS, therapists should concentrate on establishing a sustainable cooperation of parents and children during therapy, and work to achieve a low global impairment at treatment end.

**Electronic supplementary material:**

The online version of this article (10.1007/s00787-020-01528-1) contains supplementary material, which is available to authorized users.

## Introduction

Mental disorders are highly prevalent in children and adolescents [1, 2], and pose a risk to their further development [3, 4]. Despite this, only a small proportion of young people are referred to mental health care. Moreover, many of those who do receive treatment drop out prematurely (with estimates ranging from a quarter to three quarters) [5]. Besides symptom reduction, the examination of treatment satisfaction (TS) is a key indicator of the quality of health care [6]. TS has a strong face validity, serves to provide direct feedback for the therapist, and can thus help to enhance the quality of mental health care [7].

The perspectives both of the parents and of the therapist need to be considered when investigating TS in the treatment of children, while the validity of ratings by younger children remains questionable [8, 9]. Given that ratings differ considerably between these perspectives, there are increasing calls for the inclusion of multiple informants to maximize the objectivity of assessment [10–12].

Several studies have examined TS in various samples of children and adolescents with mental disorders. These studies generally examined all forms of community-based outpatient care, frequently performed in the framework of child and adolescent mental health services, with the majority investigating the parents’ perspective and revealing high TS [e.g. 7, 13, 14]. Only a small number of studies have included a therapist rating [e.g. [14–19], and generally found that the therapist-rated TS was lower than that of the parents. Studies investigating both perspectives [15–17, 19] predominantly found small to medium correlations between parent and therapist TS, again indicating the need to integrate different rating perspectives [i.e. 10, 12, 20]. These few studies investigating TS in therapist rating examined only specific mental disorders (such as ADHD and/or conduct disorder, eating disorders) rather than a broad spectrum [15, 18], included only small sample sizes [15, 18] (*n* = 41, *n* = 53), examined therapist TS after inpatient treatment [15, 16], or included only adolescent patients [15, 16, 19]. Therapist-rated TS following child treatments, by contrast, has not yet been investigated.

To optimize TS and thus reduce treatment dropouts, it appears to be crucial to examine not only TS per se but also factors that influence TS [21]. Few studies have examined correlates of TS in clinical samples of children and adolescents with mental disorders, their findings are briefly summarized below. Studies examining the relation between patient-related variables and TS (mostly parent ratings) have yielded mixed findings: While some revealed a higher parent-rated TS following treatments of younger children [22, 23] and girls [10], others were unable to find this relation [7, 13, 19, 24, 25]. Only one study investigated the association between therapist-rated TS and patient-related variables (age or gender) in adolescents [19] and did not find any significant associations. In sum, findings on patient-related variables are inconsistent and mostly rely on parent ratings. To the best of our knowledge, so far, no study has investigated the relation between therapist-rated TS and patient variables in children.

Several studies have examined the relation of TS with socio-demographic and socioeconomic variables (e.g. parents’ educational level, income, ethnicity), but these variables seem to be at best weakly related to TS [e.g. 10, 13, 19, 26].

Moreover, several studies have investigated the potential influence of mental disorder characteristics (type of mental disorder, symptom severity, symptom improvement) on TS. Bjorngaard et al. [23] found that parents of children with externalizing disorders were less satisfied with treatment compared to parents of children with internalizing disorders, while other studies did not find any relation between mental disorder and parent-rated [7, 27] or therapist-rated TS following adolescent treatments [19]. The relation of mental disorder characteristics and therapist-rated TS following treatment of children has not yet been investigated [19]. Studies investigating parent-rated symptom severity and TS revealed that low parent-reported symptom severity at treatment onset was related to high parental TS at the end of the treatment [28], while high parent-reported symptom severity at treatment end correlated with low parent- and therapist-perceived TS [17, 29]. Brestan et al. [30], by contrast, did not find any significant associations at all. To summarize, the few studies to examine the relation between TS and symptom severity had small sample sizes, mostly focused on adolescents, and yielded inconsistent results.

Studies focusing on symptom improvement during treatment found positive relations between parent- or therapist-rated TS and symptom improvement [17, 30–32], with the exception of the studies by Marriage et al. [16] and Garland et al. [7], which revealed no correlation at all.

Some researchers examined the relations of TS with symptom improvement during treatment and with symptom severity at the end of treatment. In a sample of children, Brestan et al. [30] found that parent-rated TS was more closely linked to symptom improvement than to symptom severity at post-treatment. By contrast, Mattejat and Remschmidt [17] found a higher correlation of parent TS with symptom severity at post-treatment than with the symptom improvement during treatment. Moreover, the latter authors reported higher correlations of therapist TS with symptom improvement than with symptom severity at treatment end. However, in a regression analysis by Viefhaus et al. [19], symptoms at treatment end emerged as significant correlates of TS in both therapist and parent rating. It is important to keep in mind that the aforementioned studies mostly relied on correlations. Turchik et al. [22] employed multivariate analyses and found that only 3% of the variance in parent-reported TS was explained by changes in functioning and problem severity after controlling for age and gender. In sum, few studies have examined the relation between symptom improvement and TS in samples receiving various forms of community-based outpatient treatment, and have yielded conflicting findings.

Some research groups investigated treatment characteristics in terms of treatment duration or number of sessions as further factors potentially related to TS. While some studies showed a positive relation of treatment duration [23] or a number of sessions [7] with parental TS, others did not [16, 25, 27]. Two studies investigated the impact of treatment frequency: Measelle et al. [27] reported that a greater number of contacts per month led to significantly better parental TS, while another study [10] found no influence of the actual frequency of therapy sessions, but did reveal that the satisfaction with the frequency of the therapy sessions correlated significantly with overall TS. Only one study investigated therapist ratings within a clinical sample of adolescents, revealing no relation to the number of treatment sessions [19]. This relation has not been investigated in a sample of children.

Regarding other treatment characteristics, in a sample of children and adolescents, Kapp et al. [10] investigated the relation of parent-rated TS with variables concerning the approach to treatment at outset, the organizational friendliness and the organization of therapy. The results revealed that parental TS was associated with the frequency of sessions, feeling reassured at the first appointment, and having sufficient time to ask questions. Holmboe et al. [26] found that accessibility and involvement of the parents in treatment explained most of the variation in parent TS. To date, only one study has examined other specific treatment characteristics: In a large clinical sample of adolescents, Viefhaus et al. [19] found that the number of parent-/family-focused CBT interventions, good prognosis for the overall situation, and good cooperation of parents and patients were predictors of parent-rated and therapist-rated TS. Although the results on treatment characteristics provide first hints on the potential importance on TS, most studies relied on variables of treatment intensity, while other treatment characteristics have rarely been investigated (e.g. number and type of family- or patient-focused interventions, additional pharmacotherapy), and their role in the treatment of children has not been investigated at all. Only a very small number of studies investigating TS with sufficient methodological quality (i.e., large sample sizes, description of treatment ingredients, multivariate analyses) have been published so far.

Turchik et al. [22] investigated parental TS within a large sample (*n* = 3860) of adolescents aged 12–18 years (*M* = 14.82). Only a very limited amount of variance (3%) was explained by age (younger age predicted higher TS) and by the perceived change in symptom severity and functioning (higher change was correlated with higher parental TS).

In contrast, in a large sample of children and adolescents (parent-rated subsample *n* = 770, children’s age *M* = 9.9, SD = 4.2), Kapp et al. [10] conducted hierarchical linear regression analyses on parental TS, and found that a large proportion of the variance (38.2%) was explained by the following factors: gender (higher TS in girls), satisfaction with the frequency of sessions, feeling reassured at the first appointment, and sufficient time for questions. Likewise, Holmboe et al. [26] examined parental TS in a large sample of children and adolescents (*n* = 7906, age *M* = 11.3, SD = 3.22). Whereas only a small percentage of the variance was explained by demographic, clinical and socio-demographic variables, a large amount of variance was explained by accessibility and involvement of the parents (total explained variance 19.9–42.5%).

The study by Viefhaus et al. [19] is the only study to have investigated both parental and therapist TS in a large sample of clinically referred adolescents. The authors reported that 59.4% of the variance was explained in therapist rating and 20.3% in parent rating, with mental disorder characteristics (parent- and patient-reported symptoms at post-treatment) and treatment variables (especially cooperation of patients and parents as rated by therapists) explaining most of the variance.

While these results illustrate the need to take a closer look at these factors (especially treatment variables), given the lack of studies with sufficient methodological quality, we do not yet know whether such findings can be generalized to child therapies.

To summarize, the literature on TS is inconsistent, and several limitations need to be considered when interpreting the various study findings. First, most studies rely on parent ratings [e.g. 10, 26] and very few have investigated TS on the basis of parent and therapist rating perspectives. Second, the studies only included small sample sizes [e.g. 7, 13, 15, 28, 33] (range *n* = 41–180). Third, the intensity and modality of treatments were often reported very poorly or not reported at all [e.g. 10, 22, 26]. The assessment of TS was therefore presumably based on diverse, heterogeneous forms of counseling and treatment modalities. Fourth, previous studies have investigated very heterogeneous age groups. The very few studies to include therapies of younger children examined a broad age range [10, 13, 18, 23, 24, 26, 34, 35], and no study to date has examined TS in a sample of child therapies. This would be of particular importance, as parents of younger children are more involved in the therapy process than parents of adolescents, and might differ with regard to TS. Moreover, various different analytical strategies and instruments were employed. Finally, most studies used bivariate correlations, and there are first indications that the relation between the studied variables and TS is reduced when they are examined in multivariate analyses. The sometimes inconsistent findings may at least partially be explained by all of these shortcomings. To the best of our knowledge, only one study has investigated TS from different perspectives following routine CBT in a large sample of clinically referred youth with mental disorders. This recent study by our own research group [19] addressed some of the aforementioned limitations, examining a large sample (*n* = 965) of adolescents aged 11 to 20 years following routine outpatient cognitive-behavioral therapy. Patient, parent and therapist ratings were assessed and potential predictors of TS were analyzed using regression analyses. Most of the variance in TS (parent rating *R*^2^_adj._ = 0.203, patient rating *R*^2^_adj._ = 0.322, therapist rating *R*^2^_adj._ = 0.594) was explained by mental disorder characteristics (parent- and patient-reported symptoms at post-treatment) and treatment variables (especially cooperation of patients and parents as rated by therapists), whereas patient-related or socio-demographic variables did not emerge as relevant predictors of TS.

The current study sought to add important knowledge to the research field by investigating TS rated by parents and therapists in a large sample of clinically referred children with mental disorders, who were treated with outpatient routine CBT. Specifically, we aimed to (1) describe the degree of TS as perceived by parents and therapists, and (2) explore correlates of parent- and therapist-perceived TS using multivariate analysis strategies.

## Methods

### Procedure

The inclusion criteria for this therapy study were as follows: age between 6;0 and 10;11 years, a diagnosis of a mental disorder according to ICD-10 criteria, ability to attend treatment appointments once per week, and an overall positive prognosis for outpatient treatment based on clinical judgment (e.g., no indication for other types of therapy or necessity of more intense treatment such as inpatient therapy). We excluded patients who had only received diagnostic assessments followed by brief counseling (fewer than 10 appointments overall). The study was approved by the Ethics Committee of the University of Cologne, and all parents who participated in the study provided written informed consent.

Children were referred by other inpatient or outpatient units of the University of Cologne or other hospitals, by private psychiatric practices or psychotherapeutic practices, or directly by their parents. A 1–2-h consultation appointment with the licensed child and adolescent psychotherapists (one of the authors) who were also accredited supervisors (with two exceptions: P. V., D. P.) served the purpose of examining eligibility for treatment and providing information about the treatment. We assessed eligibility for the study during the 1–10 weeks before the start of the treatment, and participants were consecutively included in the study. The first assessment (pre-assessment) took place within the first five treatment sessions and included a set of standardized questionnaires in parent and therapist rating. The second assessment including the examination of TS (post-assessment) occurred at the end of the treatment, and again encompassed ratings from parents and therapists.

A total of 1802 patients aged 6–10;11 fulfilled the entry criteria and were included in the study between January 2006 and March 2015. By March 2015, 1317 patients had completed their appointments, of whom patients with fewer than ten appointments in total (*n* = 253) were excluded. Therefore 1064 patients had received at least ten treatment sessions (100%). Complete assessments were available for *n* = 795 (75%) children, and we used this sample for the main analysis.

### Measures

#### Diagnostic interviews

All clinical diagnoses were based on clinical examination, employing the clinical rating scales of the DISYPS-II (Diagnostic System for Psychiatric Disorders in Children and Adolescents), a German semi-structured clinical interview based on the diagnostic criteria of DSM-IV and ICD-10 [DISYPS-II; 36]. Acceptable to excellent internal consistencies (ranging from *α* = 0.69 to 0.95) have been found in studies in the field and clinically referred samples of children and adolescents, and moderate correlations have been found between clinical ratings based on parent and adolescent interviews [37].

#### Treatment satisfaction

The “Therapy Evaluation Questionnaire” (TEQ) [14] in parent and therapist rating was used to measure TS at treatment end. The TEQ is a German-language questionnaire consisting of 21 items in parent rating and 26 items in therapist rating. Response options range from 0 (‘poor’) to 4 (‘excellent’). Items belong to the following subscales: two subscales in parent rating (‘treatment success’ and ‘course of treatment’) and five subscales in therapist rating (‘treatment success regarding the patient’, ‘treatment success regarding the family’, ‘patient cooperation’, ‘cooperation of the mother’, and ‘cooperation of the father’). A mean score for each rater can be calculated by summing up the item scores and dividing by the number of items. The questionnaire has shown satisfactory internal consistencies (Cronbach’s alpha *α* ≥ 0.80 for most scales); test–retest reliability (17 months) lay at *r* = 0.77 in parent rating and *r* = 0.74 in therapist rating [14]. In our sample, the internal consistencies were *α* = 0.92 in parent rating and *α* = 0.94 in therapist rating. The scale scores can be interpreted as completely unsatisfied (0 ≤ *x* ≤ 0.5), predominantly unsatisfied (0.5 < *x* ≤ 1.5), partly satisfied (1.5 < *x* ≤ 2.5), predominantly satisfied (2.5 < *x* ≤ 3.5), and completely satisfied (3.5 < *x* ≤ 4.0) [14].

#### Emotional and behavioral problems

Mental health problems were assessed at pre- and post-assessment using the German version of the parent-rated Child Behavior Checklist (CBCL; 113 items) (Achenbach System of Empirically Based Assessment; ASEBA) [38]. For the German version of the CBCL, at least satisfactory reliability and validity have been demonstrated [38–40]. Furthermore, the internal consistency of an overlapping clinic-referred sample was found to be excellent (CBCL *α* = 0.94) [37].

#### Basic documentation form

Using the standardized ‘basic documentation form’, therapists assessed socio-demographic (measured at pre-assessment; e.g. relationship of the parents, number of children in the family) and therapy data (measured at post-assessment; e.g. number of therapy sessions, type and sum of different CBT interventions chosen from a provided intervention pool) [41]. Therapists documented mental disorders on the six axes of the ‘multi-axial classification of child and adolescent psychiatric disorders in ICD-10’ [42]. Cognitive functioning was assessed using the Wechsler Intelligence Scale for Children [WISC-III; 43] or was based on clinical rating and coded on axis 3 of the multi-axial classification of child and adolescent psychiatric disorders in ICD-10 (ranging from 1—very high intelligence to 8—very severe impairment of intelligence). Global impairment (axis 6) was assessed at pre- and post-treatment, and ranged from 0 (very good functioning in all areas) to 8 (needs persistent support 24 h per day). At the end of treatment, the following clinical ratings were also included: (1) prognosis for symptom development, with scores ranging from 1 (completely cured) to 5 (poor prognosis); (2) prognosis for overall situation, with scores ranging from 1 (completely cured) to 5 (poor prognosis), and (3) cooperation of patient and parents, ranging from 1 (no cooperation) to 5 (very good cooperation).

### Outpatient treatment

The treatment took place at the university outpatient clinic of a school for child and adolescent cognitive-behavioral therapy in Germany. The therapists (*n* = 272) were postgraduate students who held a Master’s degree in psychology or education and were in the second half of their five-year child and adolescent CBT training. The CBT training includes 600 therapy sessions which are performed under the guidance of an accredited CBT supervisor (1 h of supervision every fourth therapy session). The treatment costs were covered by the German health insurance system.

### Statistical analysis

Patients with at least ten treatment sessions were included in the main analysis (*n* = 795; completer sample; treatment sessions: *M* = 43.2, SD = 19.9). Two different analyses were conducted to check for representativeness: First, we compared the completer sample (*n* = 795) to the sample which had been excluded due to missing data (*n* = 269; missing data sample treatment sessions: *M* = 31.3, SD = 18.5). Second, we compared the completer sample to the patients who were not included in the further analysis as they had attended fewer than ten appointments (*n* = 253, brief counseling sample). Comparisons were conducted for pre-assessment data, socio-demographic data, clinical ratings, and therapy characteristics using *t *tests for independent samples and Chi-square tests (in the case of dichotomous variables). We calculated the magnitude of differences using Cohen’s *d* effect sizes (*M*_incomplete _− *M*_complete_)/(SD_pooled_) [44] for continuous variables and odds ratios for dichotomous variables.

TS was described using means and standard deviations for all scales (averaged raw scores). To examine differences between the rater groups, *t* tests for dependent samples were conducted for TS ratings.

Additionally, we calculated bivariate correlations of parent and therapist ratings. To analyze overall changes during treatment according to the CBCL, we compared the total scores of the pre- to post-assessment ratings using *t *tests for dependent samples and calculated Cohen’s *d* effect sizes for continuous variables ((*M*_pre _− *M*_post_)/ SD_pre_) [44].

To examine differential effects, we conducted regression analyses for both outcome measures (TEQ total scores of TS of parents and therapists) and analyzed all variables from the basic documentation form (47 variables in total). Variables which showed high multicollinearity were excluded (variance inflation factor > 10, tolerance < 0.1) [45–48]. The predictor variables were grouped into four blocks from proximal to distal [patient variables (e.g. age), mental disorder characteristics (e.g. grouped axis 1 diagnosis, parent-rated mental disorder symptoms at post), socio-demographic variables (e.g. parents’ relationship status), and therapy variables (e.g. number of treatment sessions)]. In the first step, bivariate correlations were calculated to assess the relationship between the TEQ total scores and each potential predictor. In the second step, the significant predictors (*p* < 0.05) of the bivariate correlation were included blockwise in a hierarchical regression analysis for each outcome measure.

## Results

### Participants

Of the 795 participants, *n* = 591 were boys (74.3%) and *n* = 204 were girls (25.7%), and the age range of the sample lay between 6;0 and 10;11 years (*M* = 8;6, SD = 1;4). The majority of the patients were of average intelligence (*n* = 623, 78.4%, *n* = 114 (14.4%) above-average intelligence and *n* = 58 (7.3%) below-average intelligence). TS was mainly rated by mothers (*n* = 722; 90.2%), and no statistically significant differences between raters were found on the total scale of the TEQ (*t* test). The parents of *n* = 268 patients (33.7%) were separated and the parents of *n* = 345 patients (43.4%) reported at least one family member with at least one present or past mental disorder.

The semi-structured clinical interviews [DISYPS-II; 36] revealed the most common mental disorders to be as follows: attention-deficit/hyperactivity disorders (*n* = 175, 22.0%), hyperkinetic conduct disorder (*n* = 164, 20.6%) anxiety disorders (*n* = 110, 13.8%), conduct disorders (*n* = 75, 9.4%), elimination disorders (*n* = 63, 7.9%), other emotional disorders (*n* = 47, 5.9%), autism spectrum disorders (*n* = 46, 5.8%), tic disorders (*n* = 27, 3.4%), obsessive–compulsive disorders (*n* = 18, 2.3%) and depressive disorders (*n* = 15, 1.9%). A total of 298 participants (37.5%) had two or more mental disorders.

The global impairment, which was rated at pre-assessment based on the multiaxial system of ICD-10 [42], was as follows: *n* = 4 of the patients (0.5%) had no impairment, *n* = 30 patients (3.8%) showed satisfactory functioning, *n* = 149 patients (18.7%) had mild impairment, *n* = 335 patients (42.1%) had moderate impairment, *n* = 230 patients (28.9%) had serious impairment in at least one area, *n* = 39 patients (4.9%) had serious impairment in most areas, *n* = 6 patients (0.8%) had severe and profound impairment in most areas, and *n* = 2 patients (0.3%) needed considerable care (some danger of hurting self or occasionally fails to maintain minimal personal hygiene or gross impairment in communication).

The mean treatment duration lay at *M* = 18.37 months (SD = 9.10, range 1.9–67.3), with an average of *M* = 43.2 treatment sessions (SD = 19.9, range 10–146). The most frequent therapy interventions, as rated by the therapists at the end of treatment, are shown in Table 1. Almost all therapies included patient-centered and parent-/family-centered interventions. 59.0% of all treatments included interventions within schools or kindergartens, and 29.9% of treatments included sociotherapeutic interventions. 28.3% of the patients were additionally receiving pharmacotherapy (one substance or more, usually methylphenidate or atomoxetine).

**Table 1:** Most frequent interventions in the total sample (*n* = 795)

Intervention	Percentage of patients
Patient-focused interventions in total	99.9
Psychoeducation and cognitive methods	98.1
Token economies	93.8
Social skills training	75.0
Parent-/family-focused interventions in total	98.7
Psychoeducation and cognitive methods	98.5
Guidance to implement token economies	93.5
Methods to enhance the relationship between parents and youth	73.6
Kindergarten-/school-focused interventions in total	59.0
Psychoeducation and cognitive methods	54.7
Guidance to implement token economies	43.3
Methods to enhance the relationship between teacher and youth	16.0
Sociotherapeutic interventions in total	29.9
Counseling from social worker	14.8
Involvement of youth welfare office	7.0
Counseling from other involved therapists/physicians	9.9
Medication in total	28.3
Methylphenidate or atomoxetine	22.7
Antidepressants	1.8
Neuroleptics	1.9

### Representativeness of complete data

The representativeness of complete data (completers vs. patients excluded due to missing data) is depicted in Table 2. Significant differences emerged for most variables, with small to medium effect sizes. Completers had a higher intelligence level, and their parents were less often separated. Moreover, they showed fewer mental disorder symptoms at pre-assessment (CBCL), and therapists rated them to be less impaired at the beginning of treatment and to shower a greater improvement during therapy, and reported a better cooperation between parents and children. Moreover, completers had a longer treatment duration.

**Table 2:** Comparison of longer treatments (minimum 10 treatment sessions): cases with complete data pre- and post-assessment (*n* = 795) and those with incomplete data (*n* = 269)

	Complete data (*n* = 795)		Incomplete data (*n* = 269)		Test statistic	Statistical significance	Effect size (*d*) or odds ratio (OR)
	*M *or %	SD	*M *or %	SD		*p*	
Socio-demographic factors							
Age at start of the treatment	8.74	1.31	8.62	1.38	*t* = 1.29	0.199	*d* = 0.09
Gender: % boys	74.3		76.2		chi^2^ = 0.37	0.542	OR = 0.90
Classification of intelligence	2.92	0.55	2.97	0.50	*t* = − 1.20	< 0.05	*d* = 0.09
Relationship status of parents: % separated	34.6		45.0		chi^2^ = 0.05	< 0.001	OR = 1.55
Parent rating (pre)							
CBCL total^a^	46.05	22.33	53.80	23.33	*t* = − 4.32	< 0.001	*d* = 0.32
Therapist rating							
Global impairment (pre)	1.93	1.21	2.56	1.38	*t* = − 6.70	< 0.001	*d* = 0.52
Improvement global impairment (pre-to-post)	1.22	1.23	0.75	1.32	*t* = 5.25	< 0.001	*d* = 0.41
Cooperation of the patient (post)	3.91	0.77	3.54	0.80	*t* = 6.53	< 0.001	*d* = 0.48
Cooperation of the parent (post)	3.97	0.84	3.41	1.06	*t* = 7.86	< 0.001	*d* = 0.67
Number of treatment sessions	43.17	19.93	31.29	18.46	*t* = 8.60	< 0.05	*d* = 0.62

^a^Parent rating: complete data of *n* = 795 cases were compared to *n* = 219 incomplete cases with pre-assessment data

The comparison of completers with patients with fewer than ten appointments (brief counseling) revealed some statistically significant differences, with at most small effect sizes for socio-demographic factors. In therapist rating, mostly large effect sizes were found. Patients with complete data were younger, had a higher intelligence level, their parents were less often separated, they had more parent-reported mental health problems, and according to therapist rating, patients were less impaired at the beginning of treatment, showed a larger improvement during therapy, and therapists reported a better cooperation of parents and children (see Supplementary Table 1, available online).

### Treatment satisfaction

The TS of the parents and therapists is shown in Table 3. The overall TS was high (‘predominantly satisfied’) in both parent (*M* = 3.45, SD = 0.48) and therapist ratings (*M* = 2.80; SD = 0.60). The *t* test revealed statistically significant differences between the rater groups (*t* (794) =  − 32,33; *p* < 0.001), with therapists showing a lower TS compared to parents. Overall, 94.1% of the parents and 69.5% of the therapists were completely or predominantly satisfied. Inter-rater correlations were in the moderate range and were statistically significant (*r* = 0.48 (*p* < 0.01)).

**Table 3:** Treatment satisfaction (TS): Means and standard deviations of the TEQ scales (parent, therapist, *n* = 795)

	*M*	SD
Parent		
Success of treatment	2.96	0.76
Relationship with therapist	3.70	0.43
Total score	3.45	0.48
Therapist		
Success of treatment regarding the patient	2.86	0.77
Success of treatment regarding the family	2.36	0.73
Patient cooperation	2.99	0.68
Cooperation of the mother	3.02	0.82
Cooperation of the father	2.80	0.87
Total score	2.80	0.60

### Symptom reduction

The analysis of the completer sample (*n* = 795) yielded statistically highly significant, medium reductions of symptoms from pre- to post-assessment based on the CBCL total score (*d* = 0.62, (*t* (794) =  − 21.64; *p* < 0.001).

### Differential effects

Table 4 presents the results of the bivariate correlations between TS (TEQ total scores in parent and therapist rating) and all of the analyzed variables. The majority of the patient, mental disorder and socioeconomic variables showed no statistically significant or low to moderate correlations with TS (range between *r* =  − 0.51 and *r* = 0.40). The highest correlation was found between therapist-rated TS and therapist-rated global impairment at treatment end, and was highly statistically significant (*r* =  − 0.51), indicating that the lower the global impairment at treatment end, the higher the therapist-rated TS. Some small to moderate, highly statistically significant correlations emerged between TS and therapy variables (range between *r* =  − 0.52 and *r* = 0.68). The highest correlation was between the therapist-rated TS and the therapist-rated cooperation of the parent, revealing higher therapist-rated TS with higher cooperation (*r* = 0.68).

**Table 4:** Bivariate correlations between parent- and therapist-rated treatment satisfaction (TS, total score TEQ) and grouped predictor variables (*n* = 795)

Rating perspective—treatment satisfaction	Parent	Therapist
Patient variables		
Age	0.00	0.02
Gender (1 = boy, 2 = girl)	− 0.02	0.02
Mental disorder characteristics		
Axis 1—Grouped clinical diagnosis		
Externalizing clinical diagnoses	− 0.07*	− 0.14**
Internalizing clinical diagnoses	0.03	0.06
Both externalizing and internalizing clinical diagnoses	0.04	0.02
Other diagnoses	0.03	0.09*
Axis 3—Intellectual level	0.03	− 0.03
Axis 6—Global impairment		
Global impairment (pre)	− 0.08*	− 0.11**
Global impairment (post)	− 0.32**	− 0.51**
Improvement global impairment (pre to post)	0.25**	0.40**
Parent-rated mental disorder symptoms		
CBCL total score (pre)	− 0.14**	− 0.14**
CBCL total score (post)	− 0.26**	− 0.27**
CBCL total score—improvement (pre to post)	0.12**	0.14**
Soci-demographic variables		
Axis 5 grouped abnormal psychosocial situations		
Abnormal intrafamilial relationships	− 0.15**	− 0.25**
Familial mental disorder deviance or handicap	− 0.06	− 0.13**
Inadequate/distorted intrafamilial communication	− 0.12**	− 0.17**
Abnormal qualities of upbringing	− 0.09*	− 0.20**
Abnormal immediate environment	− 0.11**	− 0.12**
Acute life events	− 0.02	− 0.10**
Societal stressors	− 0.03	0.01
Chronic interpersonal stress associated with school work	− 0.01	− 0.02
Stress resulting from the child’s disorder	− 0.05	0.01
Total of abnormal psychosocial situations	− 0.14**	− 0.22**
Parents living together (0 = no, 1 = yes)	0.04	0.11**
Level of education (0 = no school-leaving qualification to 6 = university degree)		
Mother	0.08*	− 0.07
Father	0.05	− 0.07
Current employment (0 = no employment to 5 = full-time employment)		
Mother	0.03	0.04
Father	0.04	0.13**
Social class (1 = unskilled worker to 11 = head of larger company)	− 0.01	0.09**
Mental disorders in family	− 0.01	− 0.05
Other diseases in family	− 0.02	− 0.04
Number of siblings	− 0.03	− 0.03
Number of children in family	− 0.00	0.02
Therapy variables		
Number of specific interventions:		
Diagnostic assessments	0.05	0.02
Patient-focused CBT interventions	0.07	0.12**
Parent-/family-focused CBT interventions	0.11	0.11
School-focused CBT interventions	0.02	− 0.05
Sociotherapeutic interventions	− 0.08*	− 0.12**
Other parallel therapies (i.e. occupational therapy)	− 0.05	− 0.04
Number of all interventions	0.05	0.01
Pharmacotherapy (0 = no, 1 = yes)	− 0.01	0.06
Cooperation of the patient	0.31**	0.57**
Cooperation of the parents	0.38**	0.68**
Prognosis for symptom development (1 = very good; 5 = poor prognosis)	− 0.34**	− 0.52**
Prognosis for overall situation (1 = completely cured; 5 = poor prognosis)	− 0.29**	− 0.51**
Number of treatment sessions	0.02	− 0.01
Regular treatment end (0 = no; 1 = yes)	0.25**	0.32**

The results of the final steps of the hierarchical regression analysis for parent-and therapist-rated TS are summarized in Table 5. Collinearity was assessed and pre-treatment scores were excluded due to collinearity (CBCL total score, axis 6).

**Table 5:** Final step of the hierarchical regression analysis for the prediction of parent- and therapist-reported TS (*n* = 795), significant predictors

Rating perspective—TS	Parent		Therapist	
	*ß*_std_	*R*^2^_adj_	*ß*_std_	*R*^2^_adj_
*Patient variables*				
Mental disorder characteristics		0.120		0.254
Global impairment (post)	− 0.097*		− 0.094*	
CBCL total score (post)	− 0.109*			
*Socio-demographic variables*		0.138		0.288
Abnormal intrafamilial relationships	− 0.078*		− 0.110**	
Level of education (0 = no school-leaving qualification to 6 = university degree) Mother	0.142**			
*Therapy variables*		0.218		0.572
Cooperation of the patient	0.092*		0.240**	
Cooperation of the parents	0.240**		0.446**	
(neg.) Prognosis for symptom development			− 0.101*	
Regular treatment end (0 = no; 1 = yes)	0.096*			

*ß*_*std*_ standardized regression coefficient, *p* significance, *R*^*2*^_*adj*._cumulative adjusted *R*^2^

^*^ *p* < 0.05

^**^ *p* < 0.01

The parent-rated TS was associated with the global impairment at post (lower score correlated with higher TS), the CBCL total score at treatment end (lower score correlated with higher TS), by abnormal intrafamilial relationships (lower score predicted higher TS), by the mother’s level of education (higher level predicted higher TS) and several therapy variables: cooperation of patients and parents (higher cooperation was associated with higher TS) as well as the regular end of treatment (regular end was correlated with higher TS). Overall, these variables explained *R*^2^_adj._ = 0.218 of the variance in parent-rated TS.

The therapist-rated TS was associated with the global impairment (lower score predicted higher TS) at treatment end. Moreover, of the socio-demographic variables, abnormal intrafamilial relationships was a statistically significant predictor (lower abnormal intrafamilial relationships predicted higher TS). Of the therapy variables, the following statistically significant variables emerged: cooperation of patients and parents during therapy (higher cooperation correlated with higher TS) and the prognosis for the overall situation (better prognosis correlated with higher TS). All variables together explained *R*^2^_adj._ = 0.572 of the variance in parent TS.

## Discussion

The present study aimed to extend existing knowledge in the field of health care by investigating TS, and potential correlates thereof, in a large sample of clinically referred children following routine CBT. TS was assessed using a standardized questionnaire (TEQ) from the perspectives of parents and therapists. In addition to examining TS from these two perspectives, we investigated potential predictors in a regression analysis associated with TS. Therefore, we examined patient-related and socio-demographic data, clinical ratings and diagnosis, and parent ratings. Overall, 47 variables were grouped into 4 different categories, and variables with statistically significant correlations were included blockwise, from patient-related factors to variables relating to the treatment itself.

The overall TS was high, both in parent rating and in therapist rating. It is difficult to directly compare the present findings regarding TS following routine CBT with the results of previous studies from other research groups, as past research has examined highly diverse forms and intensities of inpatient and outpatient counseling and therapy. Nevertheless, our study did reveal similar levels of TS to previous research [14, 22].

Comparing the results of the present study with the results of our own research group’s investigation of TS in a sample of adolescents [19] on a descriptive level, the present study shows similar levels of TS (parent rating *M* = 3.45; therapist rating *M* = 2.80) to those found in the adolescent study (parent rating *M* = 3.34; therapist rating *M* = 2.69).

The inter-rater correlation of parent and therapist rating was in a moderate range (*r* = 0.48) and the therapist TS was lower (69.5% of the therapists were at least predominantly satisfied) than the parental TS (94.1% of the parents were at least predominantly satisfied). Only a small number of previous studies included the therapist’s perspective when investigating TS, and these studies examined smaller cohorts [e.g. 14–18] or different age groups [19]. Nevertheless, they revealed similar results to the present study. Thus, our findings provide support for previously found outcomes within a large sample of clinically referred children after routine CBT. Our finding of only moderate correlations between the different raters highlights the importance of including the perspectives of different raters, as called for by other researchers [i.e. 10, 11, 49, 50], as it is not possible to draw conclusions from one rater to another. In particular, the therapist appears to be a particularly critical rater, and the consideration of the therapist perspective may help therapists to reflect on and thus enhance the quality of their therapies. This is especially important given that—as our data show—treatment variables in particular are largely associated with TS, and these variables can be influenced by the therapist.

Our bivariate correlations and regression analyses concerning patient-related variables (age, gender) yielded no relations at all. Previous research findings on these variables are inconsistent, giving rise to the assumption that age and gender do not appear to have a substantial effect on the perceived TS.

Regarding the relation between mental disorders of the patients and TS, a small significant bivariate correlation was found between parental TS and clinical diagnoses of externalizing disorders, insofar as parents of children with externalizing disorders were less satisfied than parents of children without externalizing disorders. Nevertheless, when entering these variables into the multiple regression analyses, this relation was no longer found. Previous studies yielded inconsistent findings regarding clinical diagnosis. Bjorngaard et al. [23] found that parents of children with externalizing diagnoses were less satisfied than parents of children with internalizing diagnoses, whereas other studies did not find any relation [7, 27]. Therefore, future studies are needed to clarify whether the relation between TS and the type of mental disorder varies according to the type of setting or treatment employed.

The highest correlations in parent and therapist rating were found between TS and the therapist-rated global impairment at treatment end, with lower global impairment at discharge being associated with higher parent- and therapist-rated TS. A similar trend emerged with respect to the CBCL total score at discharge, with lower parent-reported mental health problems being associated with higher TS. Notably, these correlations were higher than those reflecting changes in mental disorder symptoms or global impairment during therapy. It thus appears that TS is more strongly associated with the status at treatment end than with the symptom improvement during therapy. However, when these variables were entered into a hierarchical regression analysis, in parent rating, only the CBCL total score at post and the global impairment at post remained as significant correlates, explaining 12% of the variance. These results are in line with findings on parent rating from other research groups [7, 29] and also support previous findings from our own research group [19]. In therapist rating, only the global impairment at treatment end remained a significant variable (25.4% explained variance in total). Evidently, therapists rely strongly on the extent of impairment at treatment end when rating their own TS. As such, our study extends the findings of Viefhaus et al. [19], who examined a large sample of adolescents (explained variance in total: 59.4% in therapist rating, 32.2% in patient rating, 20.3% in parent rating). Therefore, therapists should focus on reducing impairment as a prominent treatment goal. In the study by our research group examining adolescents [19], global impairment at treatment end also were associated with therapist TS. However, in this older age group, the CBCL total score at treatment end was also found to be a significant variable. When replicating these regression analyses using the externalizing and internalizing scales of the CBCL instead of the total scales, similar results were found for therapist rating. For the internalizing scale in parent rating, similar results were also found, while the externalizing scale was not a significant factor.

According to our regression analysis on socio-demographic variables, after entering patient and mental disorder variables, no noteworthy amount of variance was explained by socio-demographic factors (an additional 1.8% of explained variance in parent rating and 3.4% in therapist rating). This is comparable to the aforementioned findings of our own research group examining adolescents [19]. With regard to parent rating, we found that the fewer abnormal qualities of relationships and the higher the educational level of the mother, the higher was the parent-rated TS (both variables were statistically significant predictors but only explained a small amount of variance). In therapist rating, only the abnormal qualities of relationships emerged as a statistically significant predictor, explaining a small amount of variance. It is important to note that despite some small but statistically significant correlations, TS was not substantially related to the abnormal psychosocial situation, parents’ educational level, current occupation, or social class. These results are in accordance with findings from other research groups [e.g. 10, 13, 26].

As the relation between TS and therapy-related variables in child treatment has not been sufficiently examined so far, the present study adds important knowledge to the research field. Interestingly, variables pertaining to the therapy process accounted for a relevant additional amount of explained variance in both rating perspectives (parent rating 8.0% and therapist rating 28.4%), and several significant variables emerged. The most relevant variable in both parent and therapist rating was the parent’s cooperation during therapy, as rated by the therapist at the end of therapy (the higher the cooperation, the higher the TS). The cooperation of the patient (rated by the therapist) was also a relevant, significant factor in parent and therapist rating, as was previously found for adolescent therapies. However, in adolescents, therapist TS was better explained by the cooperation of the patients than by the cooperation of the parents [19]. This difference might be explained by the fact that parents are much more involved in the therapy process for young children, whereas the patient him- or herself is the main contact person in adolescent therapy.

Additionally, in therapist rating, the prognosis for further symptom development at the end of the treatment also emerged as a significant variable, insofar as a better prognosis was associated with higher therapist TS.

Overall, the amount of explained variance of TS varied between 22% (parent-rated TS) and 57% (therapist-rated TS), which is comparable to previous findings from our own research group regarding TS in the treatment of adolescents and to the findings of Holmboe et al. [26] and Kapp et al. [10] regarding parent-rated TS.

Our results with regard to differential effects reveal that especially in parent rating, a large amount of variance remained unexplained by the examined variables. Future studies should, therefore, address the question of whether other factors, which were not measured in the present study, affect TS. These might include, for instance, ‘feeling reassured at the first appointment’ or ‘satisfaction with the frequency of sessions’, and ‘time to formulate questions’, as suggested by previous research findings [10].

When interpreting the present findings, several limitations of the study should be taken into account. First, it is possible that there may have been an inherent selection bias in the analyzed sample, as we only included treatments with complete assessments in the analysis, and excluded treatments comprising fewer than ten sessions. According to our analyses of representativeness, excluded patients had a lower level of intelligence, their parents were more often separated, they had more parent-rated mental health problems, were more impaired at treatment begin according to therapist rating, showed a smaller improvement during therapy, and therapists reported a worse cooperation of parents and children.

As such, it is likely that our findings within the completer sample might overestimate the TS of the total sample, as was also found by Kapp et al. [10]. Moreover, another important point needs to be considered in this regard: Families most often handed their completed questionnaires to the therapist him/herself. This might have influenced parents’ answers relating to TS in the sense of social desirability, thus representing a further potential bias of the present results. Second, when interpreting the TS intercorrelations, it should be kept in mind that the parent and therapist versions of the TEQ are not completely identical with respect to items and scales. Third, when investigating the findings on differential effects, it is important to note that in therapist rating, three of the six scales of the TEQ assess the cooperation of mother, father and the young person him/herself. Obviously, this influences the relation between the cooperation rated by the therapist and the TS total score in the TEQ therapist rating. However, the finding that the two ratings of cooperation of the parents and the youngsters at the end of the treatment explained such a large amount of variance in therapists’ TS is both interesting and important. Fourth, while the therapists did receive guidance from supervisors in implementing CBT interventions, we did not formally assess treatment integrity in terms of inter-rater agreement of therapist rating or a systematic analysis of videos of treatment sessions. Furthermore, some of the patients were additionally receiving psychopharmacotherapy or sociotherapy, which presumably may also have influenced TS. Finally, although treatments were performed in a regular care setting, the therapists providing the treatment were in advanced psychotherapy training and were not fully licensed therapists in private practices. Accordingly, it cannot be fully ensured that the therapists who provided the treatments in the present study are representative of therapists who work in regular routine care settings. Future investigations should, therefore, address this question of potential differences between these two groups with respect to treatment effects, TS and predictors of TS.

Despite the aforementioned limitations, the findings of this large study on TS following routine CBT of children with mental disorders reveal a high TS in parent and therapist rating. We found that a large amount of the variance in TS was explained by mental disorder characteristics and therapy variables, while the influence of patient-related or socio-demographic variables was negligible. The clinical implications of these findings are particularly relevant given that therapists are able to influence these key factors by optimizing their individualized treatments, which may in turn also enhance therapists’ own treatment satisfaction, and help to reduce treatment dropout rates when treating children with mental disorders.

## Electronic supplementary material

Below is the link to the electronic supplementary material.

Supplementary file1 (DOCX 23 kb)
