# Supplementary material for: Parent- and therapist-rated treatment satisfaction following routine child cognitive-behavioral therapy
Source: Eur Child Adolesc Psychiatry. 2020 Apr 18;30(3):427–39. doi: 10.1007/s00787-020-01528-1 (PMC8019416; doi:10.1007/s00787-020-01528-1)
Supplement: Supplementary file 1 — Supplementary file1 (DOCX 23 kb) [file 787_2020_1528_MOESM1_ESM.docx]

Title: Parent- and therapist-rated treatment satisfaction following routine child cognitive-behavioral therapy

Paula Viefhaus, Manfred Döpfner, Lydia Dachs, Hildegard Goletz, Anja Görtz-Dorten, Claudia Kinnen, Daniela Perri, Christiane Rademacher, Stephanie Schürmann, Katrin Woitecki, Tanja Wolff Metternich-Kaizman and Daniel Walter

Correspondence to: Dr Daniel Walter, Department of Child and Adolescent Psychiatry, Psychosomatics and Psychotherapy & School of Child and Adolescent Cognitive Behavior Therapy (AKiP), Medical Faculty of the University of Cologne, Pohligstr. 9, 50969 Cologne, Germany. Tel.: +49-221-47876822, Fax: +49-221-4783962, E-Mail: daniel.walter@uk-koeln.de

**Supplementary Table 1**. Comparison of patients who had at least 10 treatment sessions (*n* = 795, longer treatment) with those with fewer than 10 appointments (*n* = 253, brief counseling)

|  | Variable | Longer treatment | | Brief counselling | | Test statistic | Statistical significance  *p* | Effect size (*d*) or odds ratio (*OR*) |
| --- | --- | --- | --- | --- | --- | --- | --- | --- |
|  |  | *M* or  % | *SD* | *M* or % | *SD* |  |  |  |
| Socio-demographic factors | |  |  |  |  |  |  |  |
|  | Age at start of treatment | 8.74 | 1.31 | 8.49 | 1.35 | *t* = 2.65 | <.01 | *d* = 0.19 |
|  | Gender: % boys | 74.3 | | 77.5 | | *chi²* = 1.01 | 0.316 | *OR* = 0.84 |
|  | Grouped intelligence | 2.92 | 0.55 | 3.05 | 0.48 | *t* = - 340 | =.001 | *d* = 0.25 |
|  | Relationship status of parents: % separated | 34.6 | | 44.5 | | *chi²* = 7.96 | <.01 | *OR* = 1.52 |
| Parent rating (pre) | |  |  |  |  |  |  |  |
|  | CBCL total^1^ | 46.05 | 22.33 | 40.98 | 21.44 | *t* = 2.45 | <.05 | *d* = 0.23 |
| Therapist rating | |  |  |  |  |  |  |  |
|  | Global impairment (pre) | 1.93 | 1.20 | 2.99 | 1.41 | *t* = - 9.50 | <.001 | *d* = 0.81 |
|  | Improvement global impairment (pre to post) | 1.22 | 1.27 | 0.12 | 0.86 | *t* = 14.22 | <.001 | *d* = 1.03 |
|  | Cooperation of patient (post) | 3.91 | 0.77 | 3.21 | 1.17 | *t* = 6.95 | <.001 | *d* = 0.72 |
|  | Cooperation of parents (post) | 3.79 | 0.84 | 2.65 | 1.34 | *t* = 12.01 | <.001 | *d* = 1.04 |

^1^ Parent rating: complete data of *n* = 795 cases were compared to *n* = 128 cases of brief counseling having pre-assessment data
